# Case Report: Fenestration embedded in large vessel occlusion at non-branching site: A catastrophic trap for mechanical thrombectomy

**DOI:** 10.3389/fsurg.2022.941557

**Published:** 2022-08-18

**Authors:** Xiaoxi Zhang, Weilong Hua, Lei Zhang, Yongxin Zhang, Yongwei Zhang, Jianmin Liu, Pengfei Yang, Xiaolong Xu, Zifu Li

**Affiliations:** Neurovascular Center, Changhai hospital, Naval Medical University, Shanghai, China

**Keywords:** mechanical thrombectomy, stroke, fenestration, stent retriever, occlusion

## Abstract

Fenestrations are rare anatomical variants characterized by division of an artery into two channels which join distally to form a single lumen. We here present two acute ischemic stroke patients with occlusion in an arterial segment with fenestration. Both occlusion sites were located at the non-branching site: one in the mid-basilar trunk and one middle cerebral artery trunk. Successful reperfusion was achieved in both patients, but angioplasty was avoided during thrombectomy procedure. The two cases establish that fenestration may be embedded in non-branching site occlusion. Surgeons should take this abnormality into account to prevent angioplasty from causing vessel rupture in the setting of fenestration.

## Introduction

Fenestrations are rare anatomical variants with incomplete fusion of embryologic vessels. They are characterized by an island window in the arterial trunk with an artery dividing into two lumens and then reforming into a single lumen (1). The two-division artery is usually smaller than a single normal artery. The two channels, each have their own endothelial and muscular layers but may share the adventitia. Vessel rupture is a catastrophic complication that may occur during mechanical thrombectomy in patients with large vessel occlusion. If the surgeons are unaware of this underlying abnormality, angioplasty within such a narrow, dividing artery may cause that vessel to rupture. We here present two thrombectomy cases complicated by fenestration to improve awareness of this deadly trap associated with mechanical thrombectomy.

### Technical notes

**Figure 1:**
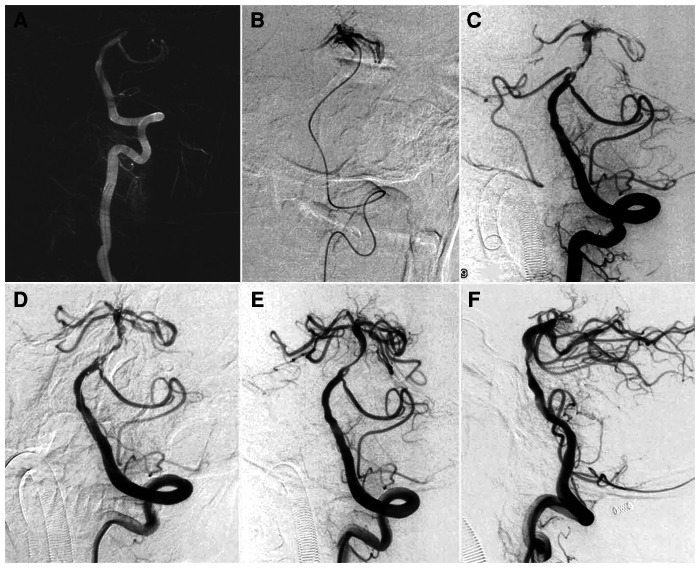
Mechanical thrombectomy for a patient with basilar artery trunk occlusion with underlying fenestration. (**A**) Left vertebral artery angiogram under roadmap showed basilar artery occlusion. (**B**) Angiogram *via* microcatheter after crossing the occlusion showed patent basilar terminus. (**C**) Microcatheter was withdrawn and a balloon was prepared to dilate the stenosis and a fenestration was located proximally to the occlusion. (**D**) Mechanical thrombectomy was performed with a Solitaire AB 4 × 20 mm which was deployed in the basilar artery. (**E,F**) After thrombectomy for one pass, complete reperfusion was achieved with antegrade flow and residue stenosis distal to the fenestration was noted.

**Figure 2:**
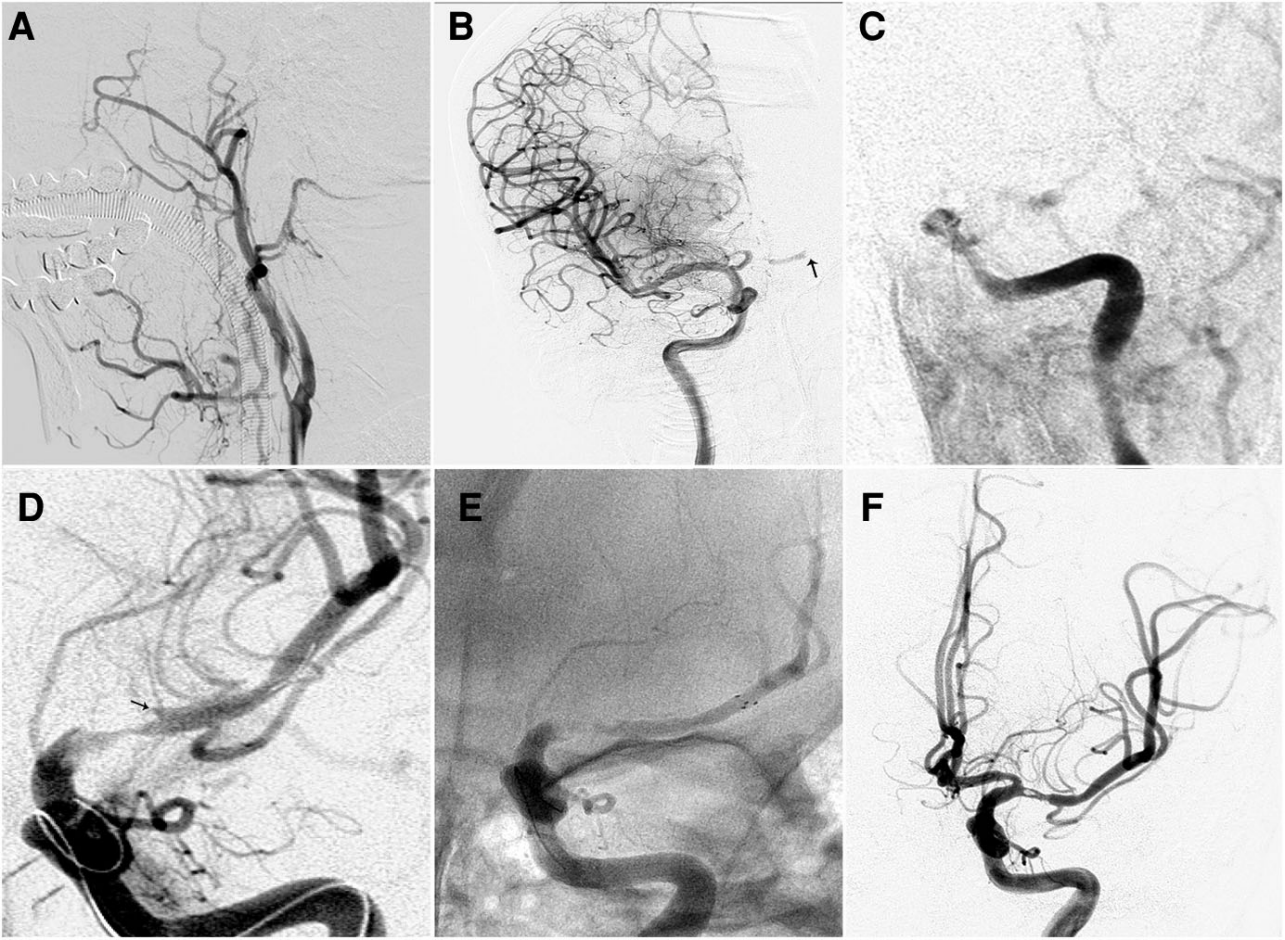
Mechanical thrombectomy for a patient with right carotid terminus occlusion and subsequent left middle cerebral artery occlusion with underlying fenestration. (**A**) Right internal carotid artery angiogram showing carotid terminus occlusion. (**B**) Control angiogram after thrombectomy showed complete reperfusion of right internal carotid artery and subsequent occlusion of left middle cerebral artery (arrow). (**C**) Left internal carotid artery angiogram confirmed occlusion of left middle cerebral artery. (**D**) After release of the Revive SE 4.5 × 22 mm, angiogram showed antegrade flow and the occluded segment highly mimicked atherosclerotic stenosis. Notably, there was a spike (arrow) toward the occluded segment. (**E**) After failure of first pass, the Solitaire 6 × 30 mm was then delivered and unsheathed. (**F**) After a second pass, complete recanalization was achieved with normal antegrade flow and fenestration was noted.

The identification of fenestration is quite important during thrombectomy, which is often observed after at least one attempt of manipulation. A spike sign, as shown in Figure 2D, should be considered for fenestration while not only irregular thrombus. A dual-track sign, as shown in Figure 1C is strongly associated with fenestration. Stent retriever is considered to be safer than aspiration with catheters, considering softer radial force. Commonly, a small size stent retriever is considered safer.

## Case reports

### Case 1

A 51-year-old man with a history of hypertension was admitted due to sudden onset of left-limb weakness and slurred speech lasting 8 h. He had a National Institutes of Health Stroke Scale (NIHSS) score of 12. Computed tomography (CT) angiography showed basilar artery (BA) occlusion.

Atherosclerotic occlusion was first considered due to absence history of embolic diseases. Mechanical thrombectomy was performed. Angiogram *via* 6F guiding catheter positioned in the left vertebral artery confirmed basilar artery occlusion. When the microcatheter (Rebar 18, Medtronic, Minneapolis, MN, US) was navigated to the distal occlusion over a microwire (Traxcess 14, Microvention, CA, US), angiogram shows mid-basilar atherosclerotic lesion with patent basilar artery terminus. Our initial intent was to perform primary angioplasty using Gateway Balloon (Boston Scientific, Natick, MA, US). However, angiography performed *via* intermediate catheter showed fenestration proximal to the lesion. Then a Solitaire FR 4 × 20 mm (Medtronic Inc., Minneapolis, MN, US) was used to retrieve the thrombus for one pass, and the basilar artery was recanalized. (Figure. 1) Dual antiplatelet therapy with aspirin 100 mg/day and clopidogrel 75 mg/day was given due to address the residual stenosis. The patient recovered without any neurological deficit as assessed at discharge one week later. Post-procedural MRI at 48 h showed massive T1 and T2 signal hyperintensity.

### Case 2

A 65-year-old woman with a history of heart valve replacement and atrial fibrillation was transferred to our hospital due to sudden onset of left-limb weakness lasting 1.5 h. The NIHSS was 18. CT angiography showed occlusion of right internal carotid artery (ICA).

Angiography confirmed the presence of occlusion in the right carotid terminus. Mechanical thrombectomy was performed with Solitaire FR 6 × 30 mm and successful reperfusion was achieved after two passes. However, the final angiogram showed left middle cerebral artery (MCA) trunk occlusion through cross-filling from the anterior commutating artery (AComA). Then, mechanical thrombectomy was first performed with Revive SE (Codman, Raynham, MA, US). After Revive SE release, angiogram showed antegrade flow and the occluded segment, which closely mimicked stenosis. After the first failure, Solitaire FR 6 × 30 mm was switched to perform second thrombectomy, and complete reperfusion was achieved for one pass. Final angiogram showed fenestration located in the proximal MCA trunk (Figure. 2). At the 3-month follow-up, the patient was hospitalized for rehabilitation with mRS 5. Post-operative CT showed slight edema and relived at 7 days CT.

## Discussion

We here describe two cases of emergent LVO and fenestration with underlying etiology of atherosclerotic and embolic diseases. A spike sign or dual-track sign, as shown in Figures 1C, 2D, should be considered for fenestration while not only irregular thrombus. Both cases showed fenestration embedded in occlusion segment at the non-branching sites, including one mid-basilar artery and one proximal MCA trunk. Although successful reperfusion was achieved using stent retriever thrombectomy, the surgical team almost attempted angioplasty before assessing the patients' anatomical features. These cases show that fenestration should be taken into account before treating cardioembolism occlusion at non-branching sites, and angioplasty should be performed with caution in the areas where fenestration is suspected.

Fenestrations are caused by incomplete fusion of embryological arteries. They segmentally divide the normal artery into two distinct channels with small diameters (2). Fenestrations are mostly located in the arterial mainstem, but they can exist in large intracranial vessels, including the AComA, BA, MCA, and vertebral artery (2). The incidence of fenestration differs at different sites, 0.1%–4.4% for MCA and 1%–5% for BA (1, 3). For patients with emergent LVO, the embolism can be set adrift only to stop at the fenestration.

The occlusion site may help indicate the presence of fenestration embedded in an occluded segment of blood vessel although differentiation from underlying atherosclerotic lesions may be difficult. Any embolism or cardioembolism is usually stuck at the bifurcation instead of at a non-branching site. When the patient has a non-branching occlusion with underlying embolic diseases, fenestration should be suspected, especially at the mid-basilar and proximal MCA trunk. Meinel et al (4) reported a case of partially occluded basilar artery fenestration at the mid-basilar trunk. Although multimodal imaging may provide indications of fenestration, differential diagnosis remains difficult when the fenestration is embedded in embolic occlusion (5, 6). According to our experiences, occlusion at the presumed trunk may be attributed to several conditions, mainly including severe atherosclerotic stenosis, early MCA bifurcation, fenestration, and pseudo occlusion due to hemodynamic stasis. After release of the stent retriever, their local configurations became slightly different. For atherosclerotic stenosis, the occluded segment is usually funnel-like, and the proximal lumen is mostly tapered and gradually returns to normal. For early MCA bifurcation, the diameter of the proximal lumen is larger than that of the distal lumen, indicating a large branch may be included in the occluded segment. For bifurcation occlusion, the point of occlusion seems to be in the mid-basilar area or MCA trunk due to hemodynamic stasis. After delayed phase on angiogram or stent retriever release, the true point of occlusion could be at the point of bifurcation. In fenestration, the occluded segment usually has a non-concentric stump and retrograde spike extending into the occlusion segment along the distal lumen as illustrated in Case 2.

Mechanical thrombectomy has been the first-line therapy for patients with emergent LVO (7, 8). For patients with suspected fenestration, an appropriate thrombectomy strategy is essential to preventing procedural complications. If fenestration was proximal to the underlying atherosclerotic stenosis demonstrated in case one, submaximal angioplasty would be feasible if performed *via* large fenestration division artery. If the fenestration was included in the stenosis, the balloon selection should be based on the caliber of fenestration division artery instead of proximal or distal normal artery. Under some circumstances, balloon angioplasty may not be suitable in patients with fenestration given the narrowness of the arteries. However, because fenestration is embedded in occlusion, its presence is difficult to comprehend. Performing angioplasty on such a narrow blood vessel could be catastrophic, especially when the condition closely mimics stenosis. Therefore, angioplasty should be performed only with caution for in cases of suspected occlusion. In cases of suspected fenestration, stent retriever thrombectomy is usually the first recommended treatment. However, there is still some risk that the stent retriever will become stuck distally to the fenestration because of caliber constriction, making it difficult to retract. Detachable stent retrievers may be a suitable option. Due to the anatomical constriction, aspiration may not work for thrombus located beyond a fenestration. In the meantime, direct advancement of aspiration catheter may result in vessel injury. Surgeons do not have enough cumulative experience using aspiration techniques for occlusion complicated by fenestration.

## Conclusion

For occlusion at the non-branching site for patients with embolic diseases, suspicion of fenestration should be raised. Stent retriever thrombectomy might be an option for the occlusion with suspected fenestration. Appropriate identification of fenestration is important to avoid angioplasty which may lead to vessel rupture for small vessel caliber around the fenestration window.

## Data Availability

The original contributions presented in the study are included in the article/Supplementary Material, further inquiries can be directed to the corresponding author/s.

## References

[1] Abdalkader M Raftopoulos C Finet P Nguyen TN Goffette P. Middle cerebral artery fenestration: Thromboembolic and hemorrhagic complications. Interv Neuroradiol. (2019) 25:644–7. 10.1177/1591019919857157 31208253 PMC6838846

[2] van Rooij SB Bechan RS Peluso JP Sluzewski M van Rooij WJ. Fenestrations of intracranial arteries. AJNR Am J Neuroradiol. (2015) 36:1167–70. 10.3174/ajnr.A4236 25655871 PMC8013011

[3] Patel MA Caplan JM Yang W Colby GP Coon AL Tamargo RJ Arterial fenestrations and their association with cerebral aneurysms. J Clin Neurosci. (2014) 21:2184–8. 10.1016/j.jocn.2014.07.005 25150765

[4] Meinel TR Pult F Gralla J Arnold M Bassetti C Jung S. Successful endovascular recanalization of a partially occluded basilar artery fenestration. Interv Neuroradiol. (2019) 25:44–6. 10.1177/1591019918793340 30092730 PMC6378526

[5] Palazzo P Ruff M Lyerly MJ Alexandrov AV. Basilar artery thrombus vs. Fenestration: a differential diagnostic challenge in acute ischemic stroke. J Neuroimaging. (2014) 24:607–9. 10.1111/jon.12069 24251913

[6] Ples H Kimball D Miclaus GD Iacob N Kimball H Matusz P Fenestration of the middle cerebral artery in a patient who presented with transient ischemic attack. Rom J Morphol Embryol. (2015) 56:861–5; pii: 561215861865. 26429187

[7] Goyal M Menon BK van Zwam WH Dippel DW Mitchell PJ Demchuk AM Endovascular thrombectomy after large-vessel ischaemic stroke: A meta-analysis of individual patient data from five randomised trials. Lancet. (2016) 387:1723–31. 10.1016/S0140-6736(16)00163-X 26898852

[8] Berkhemer OA Fransen PS Beumer D van den Berg LA Lingsma HF Yoo AJ A randomized trial of intraarterial treatment for acute ischemic stroke. N Engl J Med. (2015) 372:11–20. 10.1056/NEJMoa1411587 25517348

